# COVID-19 and herpes zoster: a call to action

**DOI:** 10.3389/fpubh.2023.1200353

**Published:** 2023-08-10

**Authors:** Mohammed Noushad, Mohammad Zakaria Nassani, Abdulaziz Samran, Mohiddin R. Dimashkieh, Mohammed Sadeg Al-Awar

**Affiliations:** ^1^College of Dentistry, Dar Al Uloom University, Riyadh, Saudi Arabia; ^2^Faculty of Applied Science, Amran University, Amran, Yemen; ^3^Faculty of Medicine and Health Science, Al-Razi University, Sana'a, Yemen

**Keywords:** herpes zoster, COVID-19, vaccine, lack of access, low-income countries

## Background

Herpes zoster (HZ), also known as “shingles,” is a vaccine-preventable disease that occurs due to the reactivation of the latent varicella-zoster virus in the sensory ganglia. Several studies have reported the reactivation of HZ in COVID-19 patients and those vaccinated against COVID-19. Currently, vaccines against HZ are not accessible globally, especially for those in LMICs.

## Main text

HZ usually presents as acute, painful vesicular rashes that can be debilitating and lead to physical disability and emotional distress. The most common complications of HZ are post-herpetic neuralgia (PHN) (occurring in 5–30% of cases), which could impact the quality of life for months or even years, and HZ ophthalmicus (HZO) (occurring in up to 10% of cases), which can lead to blindness (1). Moreover, HZ patients have an increased likelihood of developing a stroke within 30 days following infection, a risk that is mitigated with at least one zoster vaccination (2). The risk of major adverse cardiac and cerebrovascular events has also been shown to be 19% higher among HZ patients (3).

The risk of HZ increases as immune function declines, which is linked to aging and/or immunocompromising or immunosuppressive diseases or therapies (4). Other risk factors include asthma, chronic heart disease (CHD), chronic obstructive pulmonary disorder (COPD), depression, and rheumatoid arthritis (RA) (1). Currently, **two** major types of vaccines are available for HZ, namely Zostavax, which contains live attenuated varicella-zoster virus (ZVL), and Shingrix, a recombinant zoster vaccine (RZV) containing recombinant glycoprotein E in combination with a novel adjuvant (5). In the US, the Advisory Committee on Immunization Practices (ACIP) recommends the use of RZV for immunocompetent adults aged ≥ 50 years, for immunocompetent adults who have previously received ZVL, and for preferential use of RZV over ZVL due to its better efficacy (5). Despite this, HZ vaccination coverage among people aged ≥ 50 years in the US in 2019 was only 26.1% (6).

Although in the European Union, individuals 50 years of age or older are eligible for the HZ vaccine, in the United Kingdom (UK), the current shingles national immunization program covers only those aged 70–79 years. Incidentally, there is an indication of a high burden of HZ and PHN in the UK among ≥80-year-olds, especially those who were never eligible for the vaccination (7). However, based on the recommendations of the UK Joint Committee on Vaccination and Immunization (JCVI), eligibility for immunocompromised individuals will be expanded to those 50 years and over (with no upper age limit), and for the immunocompetent, it will change to 60 years of age for the routine cohort, in a phased implementation over a 10-year period, from 1 September 2023. Moreover, Shingrix (RZV) will replace Zostavax (ZV) for all newly eligible individuals (8). Realizing the demand for vaccines against HZ, Pfizer and BioNTech have recently initiated phase 1/2 study of the first mRNA-based shingles vaccine program on volunteers aged 50 years through 69 years (9).

Following the global outbreak of COVID-19, several researchers have reported the incidence of HZ and other herpesvirus infections in COVID-19 patients as well as those who have taken the COVID-19 vaccine (Table 1) (10). Herpes-like lesions were reported in COVID-19 patients as early as April 2020 (11). A large study in the US indicated that individuals with COVID-19 had a 15% higher risk of HZ than those without (12). A Spanish study on cutaneous reactions after SARS-CoV-2 vaccination indicated that 13.8% of them were due to varicella-zoster and herpes simplex reactivations, with 25.9% of those taking sick leave due to HZ (13). In Turkey, HZ has been shown to be one of the most frequently reported (24.9%) cutaneous reactions after SARS-CoV-2 vaccination (14). A UK study reported 10 patients presenting with herpetic eye disease following the COVID-19 vaccine, with 5 of them presenting with HZO (15).

**Table 1:** Selected reports of associations between herpesvirus reactivations and COVID-19/COVID-19 vaccination.

**References to zoster virus reactivation studies**	**Study characteristics**	**Findings**
Tammaro et al. (11); Herpes virus infection in COVID-19 patients.	Patients (*n =* 130) affected by COVID-19 in Sant'Andrea Hospital in Rome.	Two (1.5%) isolated herpetiform lesions. Authors speculate that they could be caused by either human herpes virus 1 (HHV-1), human herpes virus 2 (HHV-2), or VZV.
Bhavsar et al. (12); HZ in COVID-19 patients.	A retrospective cohort study of 394,677 individuals ≥50 years old with COVID-19 matched with 1,577,346 without COVID-19.	COVID-19 diagnosis in ≥50-year-olds was associated with a significantly increased risk of developing HZ. Individuals diagnosed with COVID-19 had a 15% higher HZ risk than those without COVID-19.
Català et al. (13); VZV reactivation after COVID-19 vaccination.	A cross-sectional study on patients (*n =* 405) with cutaneous reactions within 21 days of any dose of the approved vaccines at the time of the study.	VZV and herpes simplex virus reactivations accounted for 13.8% of reactions. VZV reactivation was the most reported reaction with BNT162b2, Pfizer-BioNTech, 17.2%. Out of 58 patients who took sick leave, 15 were mostly due to HZ.
Oguz et al. (14); HZ after COVID-19 vaccination.	A cross-sectional observational study of patients (*n =* 269) aged ≥18 years, who presented to 13 different dermatology clinics in Turkey after developing dermatological reactions following the administration of the COVID-19 vaccine.	HZ (24.9%) was one of the most frequent dermatological diseases after COVID-19 vaccination. A total of 60.6% of the dermatological diseases were after the administration of the mRNA vaccine, while 39.4% were with an inactivated vaccine.
Rallis et al. (15); Herpetic eye disease (HED) after COVID-19 vaccination.	A retrospective study of all patients who presented with HED within 28 days post-first dose COVID-19 vaccination. Eleven eyes (*n* = 10 patients) were included.	Six (60%) patients presented with VZV keratitis (five had concurrent other signs of herpes zoster ophthalmicus). The mean interval between COVID-19 vaccination and ocular symptoms/signs was 12.3 ± 10.3 days.

In a scoping review of 36 articles reporting vesicular reactions following vaccination against COVID-19, 11 indicated activation or reactivation of HZ (16). Moreover, a systematic review of HZ following vaccination against COVID-19 found that 5.6% of patients progressed to HZO, whereas 3.4% progressed to PHN (17). The increase in the occurrence of HZ in COVID-19 patients and those vaccinated against it indicates that potential future pandemics could also cause resurgences in HZ.

The epidemiological data on varicella-zoster virus (VZV) in Africa could be different compared to high-income countries (HICs) due to several differences between the two settings, including climate, the HIV epidemic, and malnutrition (18). The lack of access to healthcare services may also exacerbate the VZV-associated disease burden in Africa. Moreover, the significant varicella-associated morbidity and mortality in the pre-vaccine period in other settings could indicate similar findings in LMICs such as Africa. The low and high levels of VZV seropositivity in African children and adults could suggest that some primary exposure to VZV may be happening in adulthood. This could have serious implications, particularly in the context of its high-HIV burden and an aging population (19).

Reports of associations between COVID-19/COVID-19 vaccination and HZ in LMICs are very limited and mostly confined to case reports or case series. For example, a systematic review of case reports and case series in India indicated post-vaccinal HZ in 31 out of 136 COVID-19 vaccine recipients (20). Furthermore, most of the literature on HZ in LMICs, especially in Africa, is on its association and high predictive value for underlying HIV infection, with the risk being 12- to 17-fold greater. For example, a study on Ugandan HIV-positive patients indicated an annual HZ incidence of 751 per 100,000 (21). The establishment of HIV in the general population in many sub-Saharan African countries and the prevalence of up to 30% in some areas of southern Africa indicate a probably high incidence of HZ in those populations (22). Moreover, COVID-19 co-infections with HIV could further complicate matters. Therefore, making HZ vaccines available in LMICs should be a top priority.

Since RZV has several advantages over ZVL, it could be the vaccine of choice in most situations. RZV can be administered with other non-HZ vaccines such as inactivated influenza, PPSV23, and reduced antigen diphtheria-tetanus-acellular pertussis vaccines without affecting their immunogenicity (23). Studies of the adjuvanted herpes zoster subunit vaccine have demonstrated its high efficacy in the prevention of HZ in older adults as compared to ZVL (24, 25). The efficacy of RZV against HZ was shown to be high even at approximately 10 years post-vaccination, with immune responses remaining >5-fold above prevaccination levels (26). Furthermore, being a live vaccine, ZVL is contraindicated in primary and acquired immunodeficiency states/immunocompromised patients. However, RZV was shown to protect immunocompromised patients against HZ without safety concerns (27). Therefore, RZV could be the HZ vaccine of choice in LMICs due to the high incidence of HIV, especially in sub-Saharan Africa.

Currently, ZVL is licensed in just over 60 countries and RZV in only 36 countries, most of them belonging to the high-income group. Only a few LMICs have licensed the distribution of HZ vaccines, and information on their accessibility and implementation of HZ vaccination into national vaccine schedules is limited. Currently, HZ vaccines have not been licensed in any country in sub-Saharan Africa (28). Only in 2022, India, with almost 18% of the world population, became one of the few LMICs to approve RZV use. However, despite its approval and despite having a growing older adult population, it is still not available there. Recently, an expert group in India agreed that all at-risk patients >50 years of age with preexisting health conditions such as COPD, diabetes, cardiovascular diseases, chronic renal disease, and SLE should be vaccinated against HZ. Moreover, they also suggested formulating immunization guidelines for younger adults at risk due to an immunocompromised condition such as rheumatic disease, HIV, and other auto-immune diseases (29).

The high out-of-pocket cost makes HZ vaccines burdensome or inaccessible to those who lack insurance coverage. Considering the high cost of HZ vaccines, incorporating HZ vaccination into the national vaccine schedules of LMICs will not be a feasible solution there. However, the vulnerable and those in need should not be deprived of access. Cost-effectiveness studies provide information on the feasibility of recommending and implementing HZ vaccination into the vaccine schedules. The results from such studies are critical, especially in LMICs. A recent updated critical review on the cost-effectiveness of RZV vaccination against HZ indicated 15 out of 18 studies to be suggestive of being cost-effective. However, RZV was shown to be more cost-effective than ZVL (30). In another cost-effectiveness study in five Latin American countries, the introduction of RZV for older adults was shown to greatly reduce the public health burden of HZ and reduce the related doctor visits and hospitalization days (31). Since most of the countries where HZ vaccines are licensed are HICs, the majority of studies on cost-effectiveness are limited to developed economies in Europe and North America (28).

In cash-strapped LMICs, the greater public health burden related to other vaccine-preventable diseases, the lack of epidemiological data, and the lack of cost-effectiveness studies make HZ vaccines less of a priority. However, international bodies such as World Health Organization (WHO) and Gavi, in cooperation with vaccine manufacturers, should consider making HZ vaccines available globally. Considering the high incidence of HIV in LMICs, especially in sub-Saharan Africa, RZV could be the vaccine of choice. Furthermore, taking the positive association of HZ with HIV into account, one priority in LMICs, especially in sub-Saharan Africa, should be educating the public about HIV, the importance of early diagnosis, minimizing social stigma, and improving access to antiretroviral therapy (ART) for patients with HIV. ART has been shown to avert almost 169,000 HZ opportunistic infections between 1990 and 2013, in LMICs during the first 12 months of treatment (32).

In countries where HZ vaccination is cost-effective, apart from being within the recommended age group, and the immunocompromised, it should also be recommended for those with diseases such as HIV, systemic lupus erythematosus, inflammatory bowel disease, RA, CHD, COPD, and to those in the high-risk group (33). Moreover, raising awareness about HZ and the importance of seeking early medical treatment, especially in LMICs where information is lacking, is essential. Recommending bodies and policymakers should review existing guidelines and update them based on available evidence. These guidelines should be effectively communicated to healthcare practitioners on how to use and on whom to use the vaccines to prevent HZ in their patients. Since the probability of vaccine acceptance is high on the recommendation of a healthcare practitioner, they should be adequately trained to identify and recommend the vaccine to vulnerable groups, especially in LMICs. Assuming that the introduction of HZ vaccination in many LMICs into the routine vaccine schedules would not be cost-effective, apart from recommending them to only the most vulnerable, international bodies such as the WHO and Gavi should promote access to HZ vaccines there by making them available at affordable prices. We opine that vaccines should not be the privilege of only the rich.

## Conclusion

Numerous studies have established the occurrence of HZ in COVID-19 patients and those vaccinated against COVID-19. Policymakers must address this public health crisis by updating current guidelines for HZ vaccination based on the latest evidence from recent studies, and healthcare workers should be communicated on whom to vaccinate and when. Moreover, vaccines against HZ must be made accessible globally, especially in low-income countries that are left behind.

## Author contributions

MN: conceptualization. All authors contributed significantly to the preparation of the manuscript.
